# Supplementary material for: Collagen IV of basement membrane: V. Bromide-mediated sulfilimine bonds interlock the quaternary structure of NC1-hexamer of scaffolds enabling metazoan evolution
Source: J Biol Chem. 2026 Mar 6;302(5):111354. doi: 10.1016/j.jbc.2026.111354 (PMC13098435; doi:10.1016/j.jbc.2026.111354)
Supplement: Supplementary Figures [file mmc1.docx]

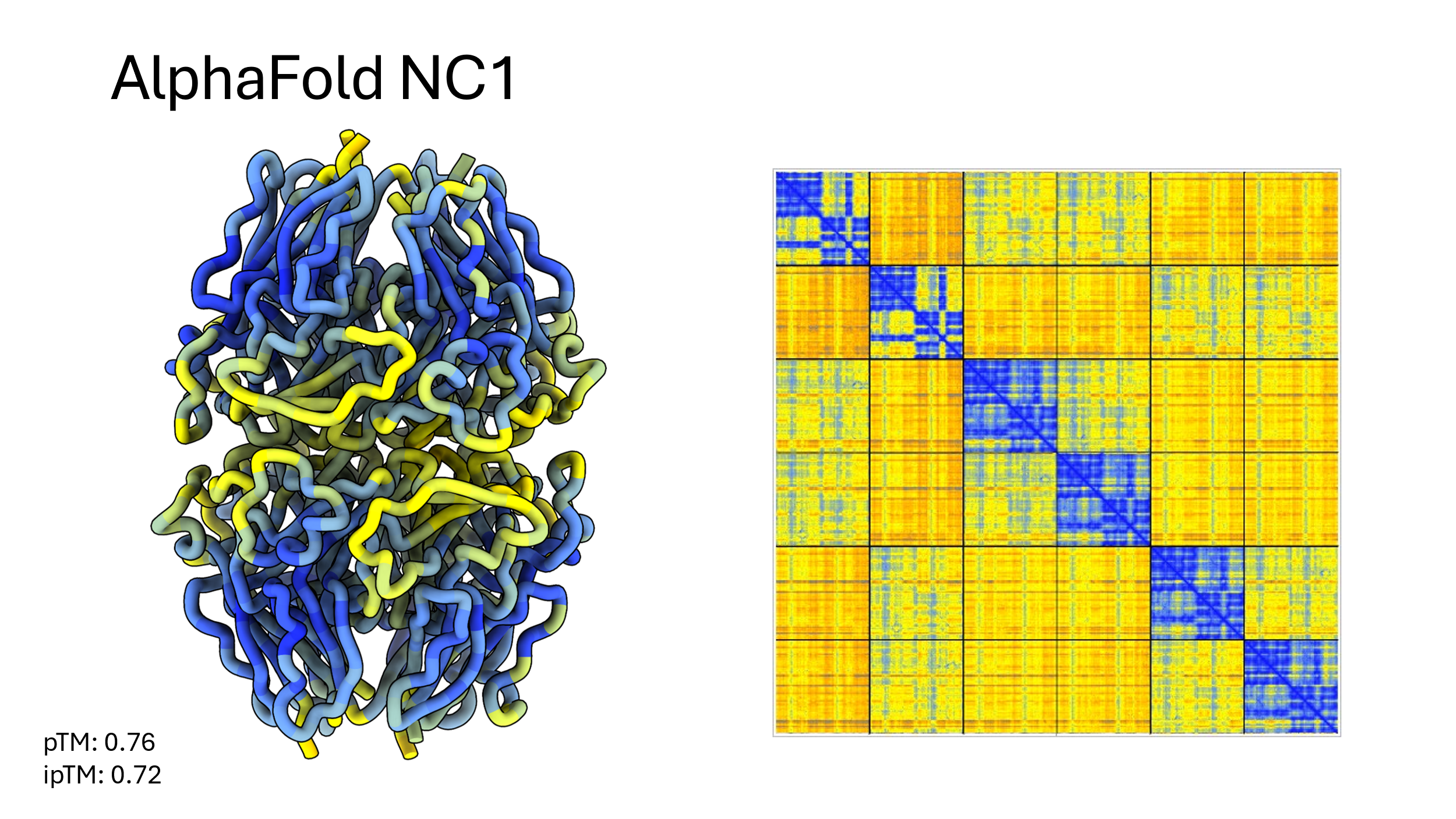


**Figure S1. AlphaFold 3 prediction of *Nematostella* Col-IV^α121^ NC1 hexamer.**

Predicted structure of *Nematostella* NC1 hexamer colored by pLDDT score, and PAE plot


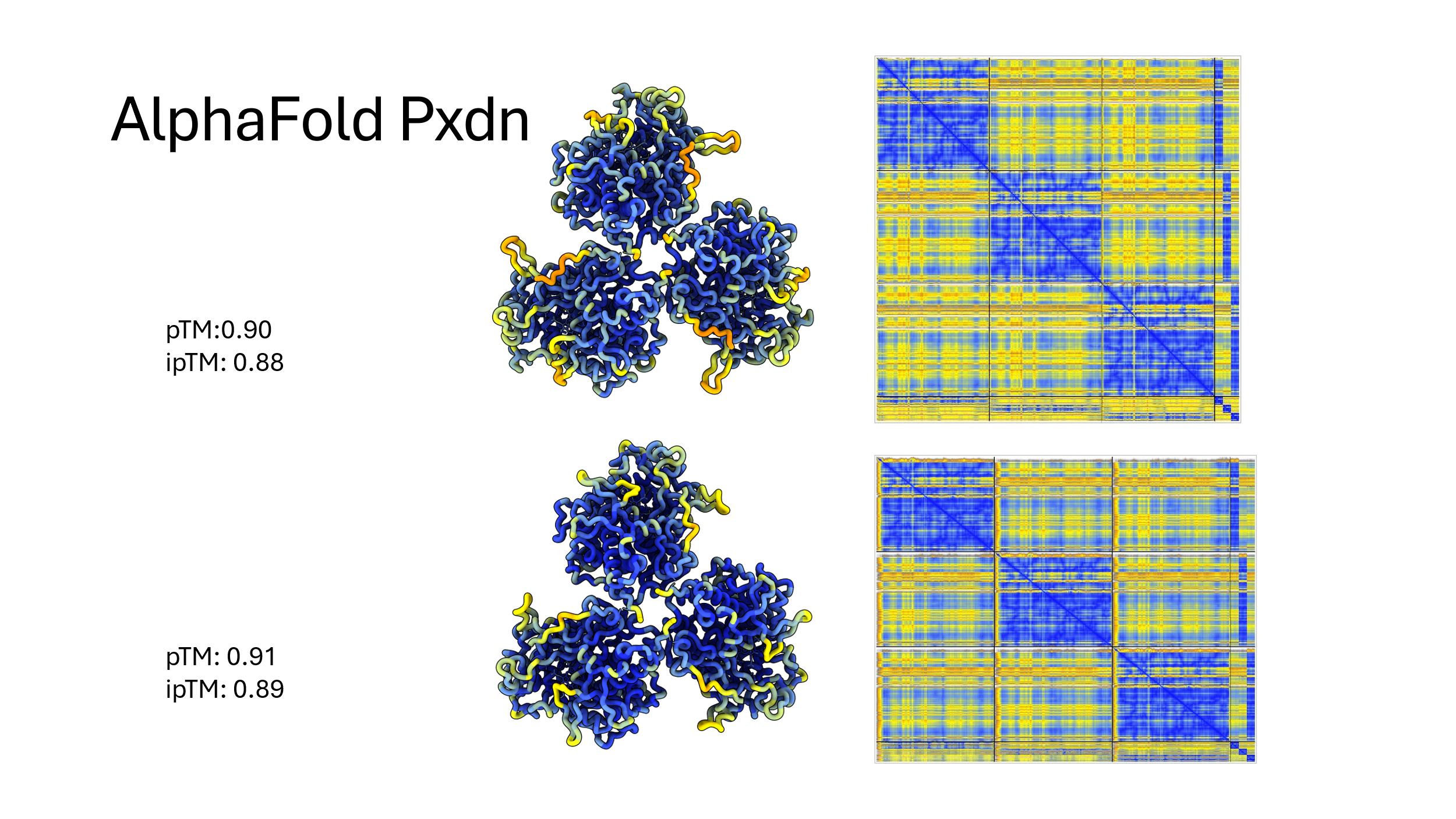


**Figure S2. AlphaFold 3 prediction of the trimeric domains of human and *Nematostella* peroxidasin.**

Prediction of Human (Top) and *Nematostell*a (bottom) peroxidasin trimers colored by pLDDT score, and PAE plots.


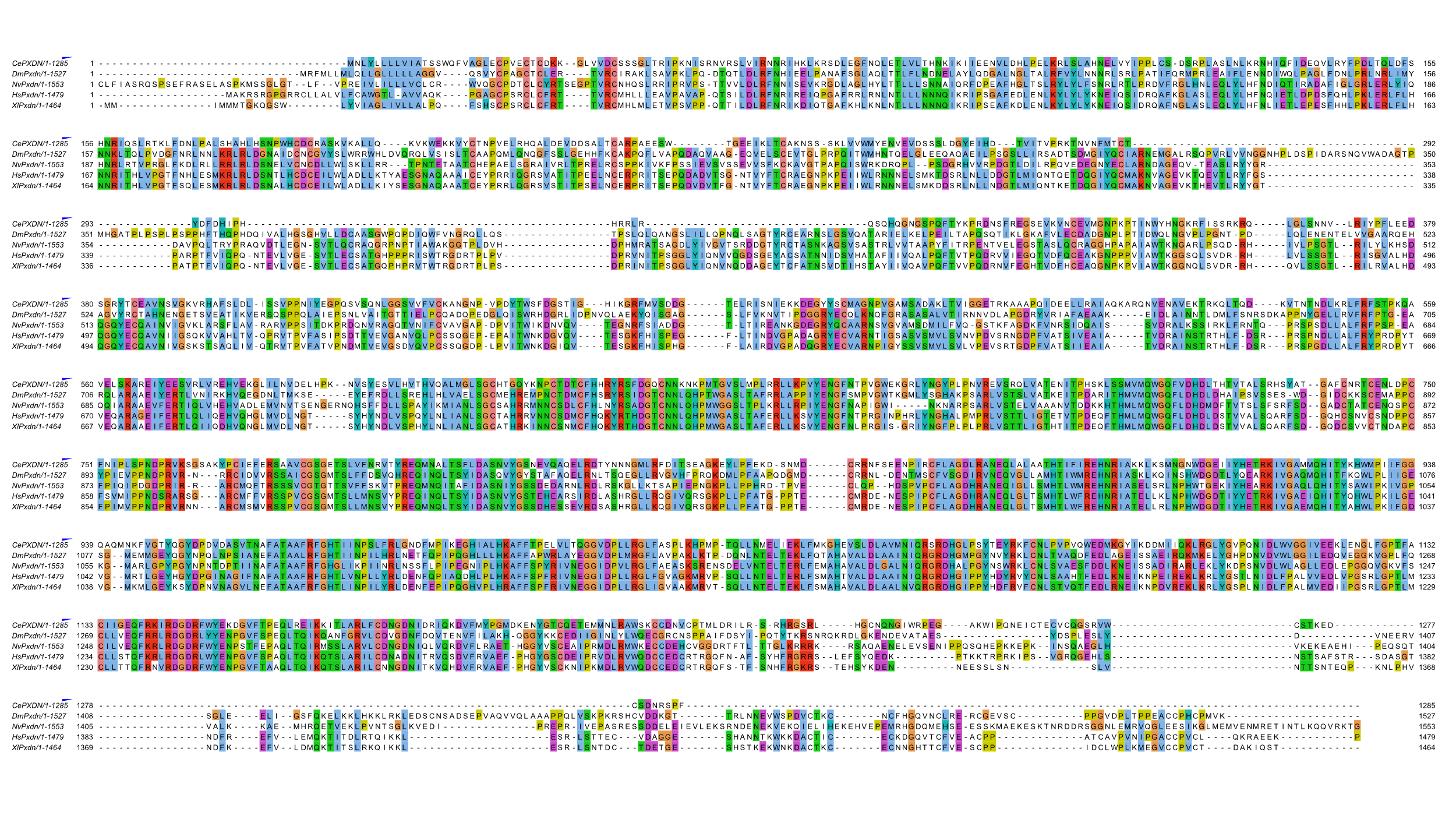


**Figure S3. Comparison of the sequence alignment of peroxidasin with other species.**

Sequence alignment of Peroxidasin from *C. elegans, D. melanogaster, N. vectensis, H. sapiens,* and *X. laevis*. This alignment was used to color peroxidasin in Figure 6 B, C, E, and F.


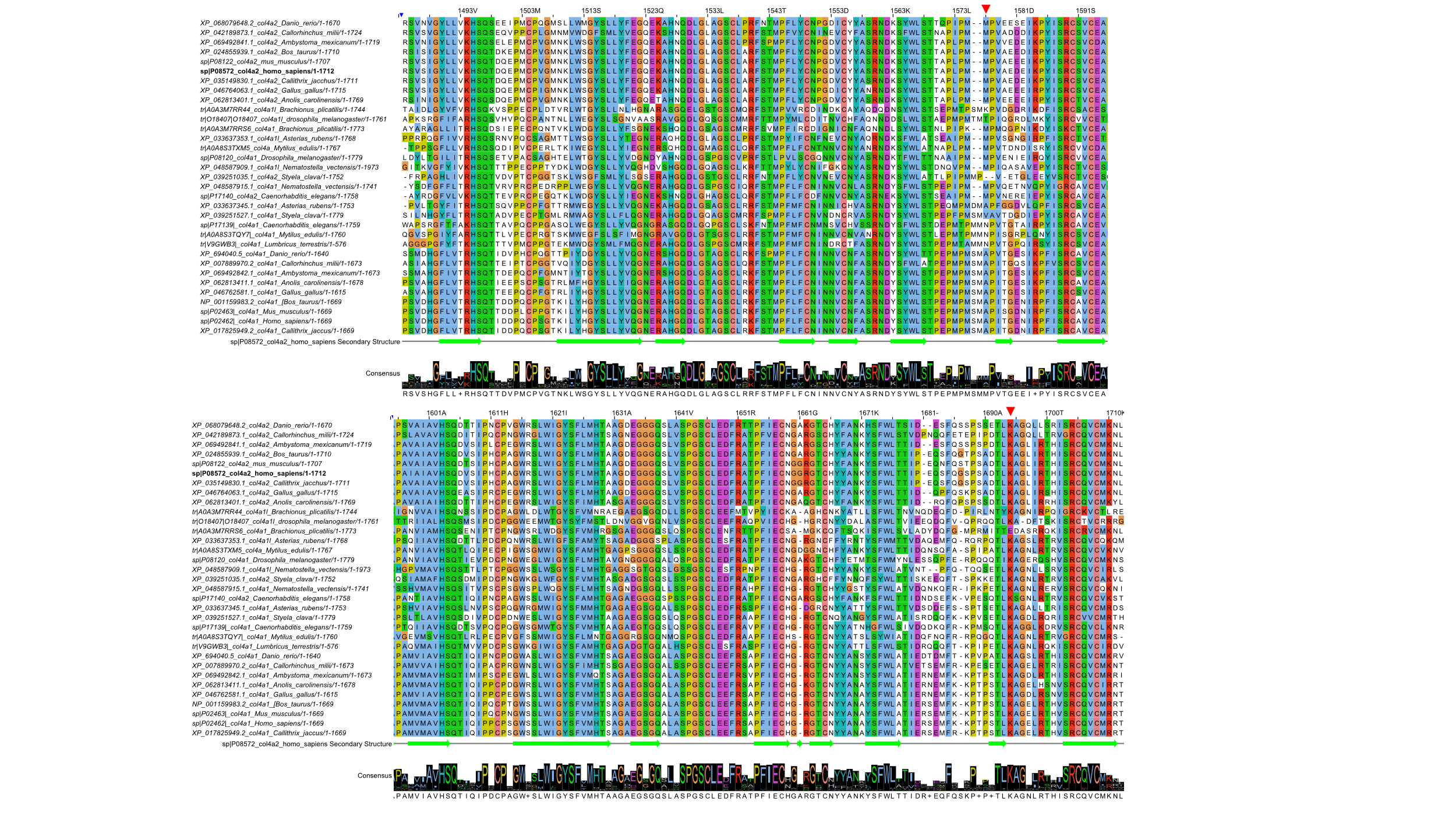


**Figure S4. Sequence alignment of Col-IV^α121^ NC1 domains.**

Sequence alignment of NC1 domains from species shown in Figure 11. Key Met and Lys residues are marked with a red arrowhead. Numbering and secondary structure (indicated below alignment) is based on *H. sapiens* Col4a2 (indicated in bold).
